# Independent validation of circulating microRNAs as biomarkers in a case-control study of adolescents with type 1 diabetes for more than 8 years

**DOI:** 10.1371/journal.pone.0343117

**Published:** 2026-02-23

**Authors:** Diana Swolin-Eide, Auste Pundziute Lyckå, Gun Forsander, Daniel Novak, Johannes Grillari, Andreas B. Diendorfer, Matthias Hackl, Per Magnusson

**Affiliations:** 1 Department of Pediatrics, Institute for Clinical Sciences, Sahlgrenska Academy, University of Gothenburg, Gothenburg, Sweden; 2 Department of Pediatrics, Sahlgrenska University Hospital, Gothenburg, Region Västra Götaland, Sweden; 3 Ludwig Boltzmann Institute for Traumatology, Research Center in cooperation with AUVA, Vienna, Austria; 4 Austrian Cluster for Tissue Regeneration, Vienna, Austria; 5 Institute of Molecular Biotechnology, BOKU – University, Vienna, Austria; 6 TAmiRNA GmbH, Vienna, Austria; 7 Department of Biomedical and Clinical Sciences, Linköping University, Linköping, Sweden; 8 Department of Clinical Chemistry, Region Östergötland, Linköping, Sweden; University of Diyala College of Medicine, IRAQ

## Abstract

MicroRNAs (miRNAs) are epigenetic regulators of gene activity. Analysis of circulating miRNAs enables minimal-invasive studies of disease mechanisms and identification of novel disease biomarkers. The aim of this case-control validation study was to investigate previously identified circulating miRNAs in Swedish adolescents with long-duration (8.0–16.5 years) type 1 diabetes (T1D), and healthy matched controls to confirm their utility as biomarker candidates to diagnose and monitor the progression of T1D. Quantitative PCR analysis of 23 previously reported miRNAs was performed in 24 T1D and 24 control individuals. Body composition was assessed by dual-energy X-ray absorptiometry and peripheral quantitative computed tomography. Prospectively collected clinical data were retrieved from the Swedish diabetes quality registry. The selected miRNAs showed higher variability in both male and female T1D groups compared to controls. Statistical analysis confirmed differences for 12 miRNAs in comparison with controls, including miR-223-3p and miR-135a-5p, which previously were reported to be associated with T1D. MiR-34a-5p and miR-210-3p were positively associated with T1D duration and HbA1c (average from the last year), respectively. In conclusion, 12 previously reported miRNAs showed consistent differential expression between individuals with T1D and controls. Among these were miR-223-3p and miR-135a-5p, which are associated with cardiovascular/inflammatory disease and cancer, respectively. These findings suggest potential clinical utility of circulating miRNAs for T1D diagnosis and disease monitoring, although extended validation of the identified miRNA biomarkers in larger, independent cohorts is required to establish the necessary scientific evidence for clinical translation.

## Introduction

Type 1 diabetes (T1D) is an autoimmune disease, which is characterized by progressive loss of insulin-producing pancreatic β-cells during months or years before clinical onset [1]. There is a complex immune dysregulation in T1D which, except from environmental factors, also include genetic factors, innate immune dysfunction, and islet autoreactive cells [2]. T1D commences with multiple autoantibodies (≥2) in stage 1, added by temporally pathological glucose tolerance in stage 2, and finally clinical onset with typical symptoms of shortage of insulin in stage 3 [3]. Children diagnosed at 6 months to 18 years of age represents about half of the number of persons that are afflicted in Sweden (The Swedish National Diabetes Register, https://ndr.registercentrum.se). In this national register, the mean age at diagnosis is approximately 9 years (stage 3), which implies many decades of demanding and costly treatment in the future for the individual.

The ultimate goal is to prevent the T1D and to avoid long-term complications in those already affected by the disease. However, just one single disease-modifying therapy for all individuals facing or living with T1D is unlikely in the near future [4,5]. The key question is therefore how to stratify successful treatment for each individual at risk for developing T1D, or already diagnosed with T1D; i.e., personalized medicine, where the availability of validated miRNA biomarkers is of clinical concern.

Biomarkers as microRNAs (miRNA) have increasingly been investigated, both as markers of an arising disease but also as indications of emerging complications [6–8]. Therefore, there is an increased interest in the exploitation of miRNAs as diagnostic or prognostic biomarkers, and the number of CE-marked miRNA-based diagnostic tests are increasing [9].

We have recently published a study on circulating miRNAs as biomarkers that distinguish adolescents with long duration T1D (mean 11.1 years) from matched controls [10]. Twenty-eight of the investigated miRNAs were differently regulated in comparison with healthy controls. Several cancer-related associations were identified for the six miRNAs showing the greatest differences in plasma levels. This study is aiming for an independent validation of the earlier findings to confirm or reject miRNAs that could be of value for future exploration of complications related to long-term T1D in young people.

## Methods

All methods were carried out in accordance with relevant guidelines and regulations.

### Subjects and study design

Twenty-four adolescents aged 15.1–17.9 years, 10 females and 14 males, with a mean diabetes duration of 10.6 years (8.0–16.5 years) were included in this case-control study. These individuals were identified from the Swedish National Diabetes Register (https://ndr.registercentrum.se) and were regularly monitored at the Queen Silvia Children’s Hospital in Gothenburg, Sweden. Since 2007, all 43 pediatric clinics in Sweden prospectively report clinical follow-up visit data for children with diabetes to the Swedish National Diabetes Register. This registry is funded by the Swedish Association of Local Authorities and Regions, which is web-based since 2008, and it is estimated to include 97.5% of all children with T1D aged 0–17.99 years. The control group comprised 24 age-matched healthy adolescents, 15.1–17.9 years, 10 females and 14 males, living in the Gothenburg area. Data was collected between September 16, 2020, and May 25, 2022. Clinical data are presented in Table 1. Exclusion criteria were obesity, celiac disease, hypothyroidism, metabolic, skeletal and inflammatory diseases, breastfeeding and pregnancy. Blood glucose and HbA1c levels were not measured in healthy controls to confirm normal glucose tolerance; however, the estimated prevalence of undiagnosed diabetes in the study region is low (approximately 0.05%).

**Table 1:** Clinical data of subjects with T1D and healthy matched controls.

	T1D (n = 24)	Controls (n = 24)	P-value
Age (years)	15.9 (15.1–17.9)	16.0 (15.1–17.9)	0.73
Sex (females/males)	10/14	10/14	
BMI (kg/m^2^)	22.5 (2.5)	21.4 (2.7)	0.09
***DXA measurements***			
Left leg fat mass (%)	29.7 (12.5–44.1)	23.8 (14.1–40.6)	0.16
Trunk fat mass (%)	24.0 (8.4–49.6)	17.6 (7.8–40.2)	0.18
Total body (less head), fat mass (%)	27.8 (10.2–48.5)	19.6 (10.7–42.2)	0.22
Total body (less head), lean mass (g)	43,399 (31,713–57,611)	42,210 (29,700–59,467)	0.84
Comorbidities (other than T1D)	Attention deficit disorder and autism (n = 1) Hypertension (n = 1) Microalbuminuria (non-diabetic (n = 1) Severe acne (n = 1)	Allergy for pollen (n = 3) Attention deficit disorder (n = 1)	
Medications (other than insulin)	Enalapril (n = 2) Contraceptives (n = 4) Isotretinoin (n = 1) Ritalin (n = 1) Melatonin (n = 1)	Antihistamine (n = 1) Contraceptives (n = 1) Medikinet (n = 1)	

For categorical variables n (%) is presented. For normally distributed continuous variables, mean (SD) are presented. For non-normal continuous data median (minimum – maximum) are presented. P-values calculated by Student’s t-test or Mann-Whitney U test.

BMI, body mass index; T1D, type 1 diabetes.

We selected 23 candidate miRNA biomarkers for investigation in this validation cohort (Table 2). Sample size for miRNA analysis was derived from power analysis, which was performed using G*Power Version 3.1.9.7. based on a Wilcoxon-Mann-Whitney test (two groups) model and utilizing the results from our previously reported discovery study to estimate the effect size d. We assumed equal group sizes (N2/N1 = 1), α error probability of 0.05, a power (1-β error probability) of 0.80, and an effect size d = 0.85, which resulted in a suggested total sample size of n = 48 (24 per group) achieving an actual power of 0.803. Because the statistical analysis was limited to univariable group comparisons without multivariable regression models (see below), diagnostics for multicollinearity such as tolerance or variance inflation factor were not applicable.

**Table 2:** List of the investigated miRNAs.

#	miRNA-ID	Selection criteria	TSI (SD); enriched tissue
1	miR-143-3p	T1D, Swolin-Eide et al. [10]	**0.89 (0.03); artery**
2	miR-17-5p		0.77 (0.04)
3	miR-29b-3p		0.79 (0.06)
4	miR-101-3p		0.71 (0.05)
5	miR-128-3p		**0.91 (0.02); brain**
6	miR-135a-5p		**0.94 (0.02); thyroid**
7	miR-192-5p		**0.92 (0.02); gastrointestinal**
8	miR-19b-3p		0.73 (0.06)
9	miR-215-5p		**0.93 (0.02); gastrointestinal**
10	miR-223-3p		**0.82 (0.03); neutrophils, platelets**
11	miR-410-3p		**0.87 (0.05); brain**
12	miR-495-3p		**0.82 (0.05); brain**
13	miR-21-5p	T1D, Margaritis et al. [11]	0.74 (0.03)
14	miR-375-3p		**0.94 (0.02); pancreas**
15	miR-126-3p		0.78 (0.04)
16	miR-181b-5p		0.78 (0.02)
17	miR-210-3p		0.55 (0.02)
18	miR-30a-5p		0.72 (0.04)
19	miR-122-5p	Liver/Metabolic disease	**0.95 (0.01); liver**
20	miR-124-3p	CNS disease	**0.95 (0.02); brain**
21	miR-1246	Inflammatory disease	0.69 (0.05)
22	miR-146a-5p	Inflammatory disease, T2D	0.68 (0.06)
23	miR-34a-5p	Cancer, senescence, T2D	0.64 (0.04)
24	miR-451a	Hemolysis indicator	**0.91 (0.02); erythrocytes**
25	miR-23a-3p	Hemolysis control	0.75 (0.04)
26	PCR spike-in	Synthetic Control	Not applicable
27	cDNA spike-in	Synthetic Control	Not applicable
28	RNA spike-in	Synthetic Control	Not applicable

Bold font indicate TSI-values >0.80.

miRNA, microRNA; T1D, type 1 diabetes; T2D, type 2 diabetes; TSI, tissue specificity index.

The current study was approved by the regional research ethics committee of the University of Gothenburg (No. 1076−18). All study participants and their parents received oral and written information prior to the study start, and written informed consent was obtained. Both case and control individuals were enrolled at The Queen Silvia Children’s Hospital, Sahlgrenska University Hospital, Gothenburg, Sweden.

### Assessment of body composition

Body composition was determined by dual-energy X-ray absorptiometry (DXA) Lunar iDXA (GE Lunar Corp., Madison, WI, USA).

### MiRNA analysis

Blood collection into EDTA plasma tubes was performed between 8:00 a.m. and 3:00 p.m., and directly placed on wet ice. Within 30 min after blood draw, EDTA plasma tubes were first centrifuged at 1000 × *g* at 4 °C for 10 min, followed by a second centrifugation step at 3,800 × *g* at 4 °C for 15 min. The supernatant (i.e., EDTA plasma) was aliquoted into nuclease-free tubes and immediately stored at −80 °C until analyses.

Plasma samples were processed as previously described for the discovery cohort [10]. Briefly, a single plasma aliquot was thawed on ice and centrifuged at 12,000 × *g* for 5 min to remove any cellular debris. For sample lysis, 200 µL of plasma were mixed with 1000 µL Qiazol to which 1 µL spike-in controls had been added (Qiagen, Germany). Following incubation at room temperature for 10 min, 200 µL chloroform were added to the lysates. After centrifugation at 12,000 × *g* for 15 min at 4 °C, 650 µL of the upper aqueous phase were obtained and mixed with 7 µL glycogen (50 mg/mL) to enhance precipitation. RNA washing and clean-up was performed using the miRNeasy mini kit (Qiagen, Germany): the aqueous phase was transferred to a column, and RNA was precipitated by adding 750 µL ethanol. Washing with RPE and RWT buffer was performed in a QiaCube liquid handling robot (Qiagen, Germany). In the last step, total RNA was eluted in 30 µL nuclease free water and stored at −80 °C until further analysis.

Reverse transcription (RT) quantitative PCR (qPCR) analysis of 23 distinct miRNAs and 5 controls was performed as previously described [12–15]. In brief, cDNA was synthesized using the miRCURY RT kit (Qiagen, Germany) where 2 µL of total RNA were used as input per 10 µL RT reaction mix. Cel-miR-39-3p was added to each RT reaction to monitor RT efficiency. PCR amplification was performed in a 384-well plate format using custom Pick&Mix plates (Qiagen, Germany) in a Roche LC480 II instrument (Roche, Germany) and miRCURY SYBR(R) Green Mix (Qiagen, Germany) with the following settings: 95 °C for 10 min, 45 cycles of 95 °C for 10 s and 60 °C for 60 s, followed by melting curve analysis. Cycle of quantification values (Cq-values) were determined using the second derivative method as provided by the Roche LC480 software. Data quality was assessed by visual inspection of spike-in control data. Hemolysis was assessed in all samples using the ratio of miR-23a-3p versus miR-451a.

### Statistical analysis

RT-qPCR miRNA data were normalized to the Cq-value of the RNA Spike-In (UniSp4) to account for analytical variability (Cq_UniSp4_ – Cq_miRNA_ = normalized deltaCq). Missing miRNA data below the detection limit were not imputed. Raw and normalized RT-qPCR data were uploaded to the NCBI Gene Expression Omnibus data repository (GSE315292). Clinical data were complete and assessed for their differences between T1D and controls using parametric t-test or non-parametric Mann-Whitney U test. Mean and standard deviation or median with range were reported depending on the data distribution. For visualization and unsupervised clustering of samples based on circulating miRNA levels, a heatmap of univariate scaled and centered Cq-values and principal component analysis were performed using the R packages pheatmap and pcaMethods using the web-based tool ClustVis 2.0 [16]. Differences in miRNA levels between T1D and controls were tested using Mann–Whitney U tests, and p-values were adjusted for multiple testing with the Benjamini-Hochberg (BH) method and reported on a significance level of 0.1 to restrict the percentage of false discoveries to ≤10%. Correlations between miRNA expression and continuous variables were investigated using Spearman correlations and adjusted for multiple testing. Tissue specificity indices (TSI) were obtained from the Human miRNA Tissue Atlas [17]. Mean and standard deviation were calculated from TSI values for raw, variance stabilized normalization, and quantile normalized microarray data.

## Results

### Registry data and body composition

Clinical data and comorbidities from the Swedish National Diabetes Register for the included individuals are presented in Table 1. The T1D and control cohorts comprised 24 individuals (10 females in each cohort) with a median age of 15.9 and 16.0 years for the T1D and control cohort, respectively. Clinical diabetes data for the T1D group are summarized in Table 3. The mean (SD) age at T1D diagnosis was 5.6 (2.7) years with a diabetes duration between 8.0 to 16.5 years. Continuous glucose monitoring was performed in 100% of T1D patients at the time of blood collection, and 21 patients (88%) were using a continuous subcutaneous insulin infusion pump. Hence, the study participants were generally well-controlled during the entire age span from the T1D diagnosis: mean (SD) glycated hemoglobin A1c (HbA1c) of the last measurement before the study was 52.9 mmol/mol (9.0), 7.0% (0.8). In line with our previous study [10], no significant differences were found between the study and control groups for body composition assessed by DXA (Table 1).

**Table 3:** Clinical diabetes data for the T1D group.

	T1D (n = 24)	
Age at diabetes onset (years)	5.6 (2.7) 5.6 (0.9; 9.4)	
Diabetes duration (years)	10.6 (2.4) 9.9 (8.0; 16.5)	
Continuous glucose monitoring (*n*)	24 (100%)	
Continuous subcutaneous insulin infusion pump (*n*)	21 (88%)	
**HbA1c according to age**	**IFCC (mmol/mol)**	**NGSP (%)**
Last HbA1c measurement	52.9 (9.0) 50.0 (39.0; 70.0)	7.0 (0.8) 6.7 (5.7; 8.6)
Last year mean HbA1c	54.1 (9.7) 51.8 (38.3; 78.5)	7.1 (0.9) 6.9 (5.7; 9.3)
HbA1c, 0–8.9 years	52.9 (8.6) 55.2 (39.0; 71.3)	7.0 (0.8) 7.2 (5.7; 8.7)
HbA1c, 9.0–13.9 years	53.7 (6.5) 54.9 (40.8; 62.6)	7.1 (0.6) 7.2 (5.9; 7.9)
HbA1c, 14.0–17.9 years	54.6 (8.9) 54.8 (38.3; 77.6)	7.2 (0.8) 7.2 (5.7; 9.3)
HbA1c, 0–17.9 years	54.7 (6.5) 55.5 (41.2; 70.0)	7.2 (0.6) 7.2 (5.9; 8.3)

Continuous glucose monitoring, real time or intermittent scanning. For continuous variables, mean (SD)/ median (minimum; maximum) are presented.

HbA1c, glycated hemoglobin A1c; IFCC, International Federation of Clinical Chemistry; NGSP, National Glycohemoglobin Standardization Program; T1D, type 1 diabetes.

### Circulating miRNA regulation in adolescents with long-duration T1D

Twenty-three candidate miRNA biomarkers were selected for investigation in this validation cohort (Table 2). Twelve miRNAs were selected based on our previous assessment of circulating miRNAs in adolescents with T1D [10], while 6 miRNAs were selected based on a 2021 meta-analysis [11] on the associations of circulating miRNAs with T1D. Two miRNAs were added to the panel due to their high tissue specificity and validation as organ disease biomarkers: miR-122-5p is a validated biomarker of chronic and acute liver disease [18], and miR-124-3p is CNS-specific and a known biomarker of acute or chronic brain disease [19–21]. Finally, 3 miRNAs were included: miR-1246 is associated with inflammation [22], miR-146a-5p was selected because of the enrichment in immune cells and association with chronic inflammation [23] and type 2 diabetes (T2D) [24], and miR-34a-5p because of the association with cancer, senescence and T2D [24].

The panel of 23 miRNAs was complemented by 5 quality control assays (Table 2). The RNA spike-in control showed low variability across all 48 samples, which confirms consistent total RNA recovery during RNA isolation (S1A Fig). The cDNA and PCR spike-ins show low variation, which indicates absence of RT-qPCR inhibitors and constant RT-qPCR efficiency (S1B Fig). The hemolysis index was estimated using the delta Cq-value of miR-23a-3p – miR-451a, which was homogeneously distributed across samples (S1C). The qPCR data were normalized to the RNA spike-in control to replicate the normalization approach applied during the discovery study [10].

**Fig 1:**
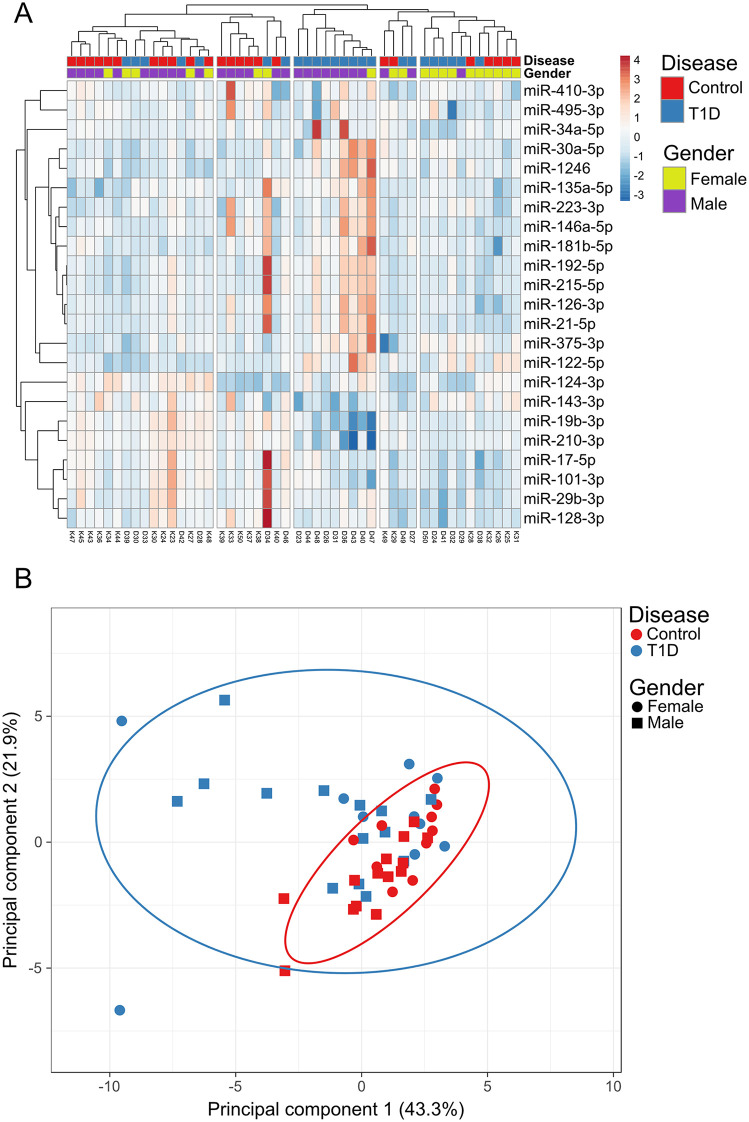
Unsupervised clustering analysis. Normalized dCq-values for 23 miRNA biomarker candidates were used to generate expression heatmaps with **(A)** Euclidean distance based clustering and (**B**) principal component analysis (PCA). Circles in the PCA plot indicate 95% confidence intervals.

An unsupervised analysis was performed to investigate plasma miRNA levels and association between T1D and controls. Based on hierarchical clustering, using Euclidean distance, we identified 5 clusters corresponding to either a mix of T1D and controls, or samples from individuals with T1D (Fig 1A). We did not observe clustering according to gender within or across the disease groups. Principal component analysis (PCA) identified tight clustering of male and female controls, which suggests that the variability in miRNA profiles is low in the control group. The male and female T1D groups showed larger variability in their miRNA profiles, which is indicated by larger 95% confidence intervals in the PCA plot (Fig 1B). Based on the number of samples outside the confidence interval, higher variation was present among males with T1D in comparison with females.

**Fig 2:**
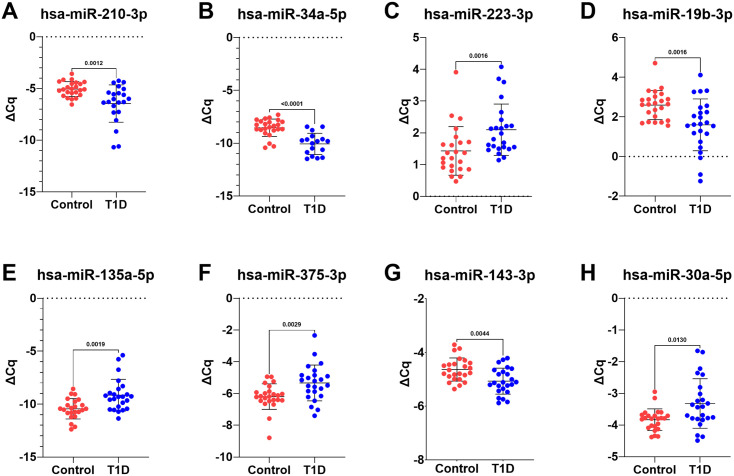
Eight miRNAs demonstrate reproducible significant differences in plasma of young adolescents with T1D. Panels A-H show the normalized dCq-values for 8 miRNAs with adjusted p-values <0.05. Wilcoxon rank sum tests were performed. Unadjusted P-values are indicated on the plot. Controls, n = 24; T1D, n = 24.

Next, we performed statistical analysis to identify miRNAs with significant differences between T1D and controls. In total, 12 miRNAs showed an adjusted p-value of <0.1 (Table 4). Of these, 7 had previously been reported in our discovery study of which miR-223-3p and miR-135a-5p showed the lowest adjusted p-value (0.012) and up-regulation as observed in the discovery cohort. Similarly, miR-192-5p and miR-215-5p (both enriched in the GI) were significantly up-regulated in T1D patients, thus confirming the discovery study results. Four out of the 6 miRNAs, which had been reported in the meta-analysis by Margaritis et al. [11], were found to be either significantly down-regulated (miR-210-3p) or up-regulated (miR-375-3p, miR-30a-5p, miR-181b-5p). Finally, miR-34a-5p was also present in the top list of miRNAs (adjusted p-value 0.012, log_2_FC –0.59). Fig 2 illustrates the plasma levels for the top 8 miRNAs based on the adjusted p-values.

**Table 4:** Statistical analysis of the miRNA results.

miRNA-ID	Sample size (Control; T1D)	Mean dCq-value	Log2FC (T1D/Control)	P-value (Mann-Whitney)	Adjusted p-value (BH)
miR-210-3p**	**24; 22**	**–5.72**	**–1.40**	**0.0012**	**0.012**
**miR-34a-5p**	**24; 19**	**–8.80**	**–0.59**	**0.0017**	**0.012**
miR-223-3p*	**24; 24**	**1.76**	**0.67**	**0.0019**	**0.012**
miR-19b-3p*	**24; 24**	**2.09**	**–1.00**	**0.002**	**0.012**
miR-135a-5p*	**23; 24**	**–9.79**	**1.29**	**0.002**	**0.012**
miR-375-3p**	**24; 24**	**–5.77**	**0.86**	**0.004**	**0.015**
miR-143-3p*	**24; 24**	**–4.85**	**–0.43**	**0.005**	**0.019**
miR-30a-5p**	**24; 24**	**–3.58**	**0.51**	**0.014**	**0.043**
miR-192-5p*	**24; 24**	**–2.56**	**0.78**	**0.028**	**0.078**
miR-128-3p*	**24; 24**	**–6.52**	**–0.33**	**0.036**	**0.080**
miR-181b-5p**	**24; 24**	**–7.58**	**0.68**	**0.037**	**0.080**
miR-215-5p*	**24; 24**	**–3.16**	**0.75**	**0.049**	**0.094**
miR-126-3p**	24; 24	0.12	0.56	0.062	0.11
miR-21-5p**	24; 24	1.64	0.67	0.11	0.18
miR-29b-3p*	24; 24	–3.96	–0.28	0.20	0.29
miR-101-3p*	24; 24	0.59	–0.27	0.21	0.29
miR-17-5p*	24; 24	0.53	–0.15	0.21	0.29
miR-1246	24; 24	–2.88	0.41	0.31	0.39
miR-146a-5p	24; 24	–3.08	0.38	0.32	0.39
miR-124-3p	24; 22	–12.27	–0.54	0.33	0.39
miR-122-5p	24; 24	–3.02	0.39	0.37	0.42
miR-495-3p*	24; 21	–9.82	–0.28	0.58	0.63
miR-410-3p*	23; 22	–9.44	–0.09	0.88	0.88

Bold font indicate adjusted p-values of <0.10.

BH, Benjamini-Hochberg; miRNA, microRNA; T1D, type 1 diabetes.

*Swolin-Eide et al. [10].

**Margaritis et al. [11].

**Fig 3:**
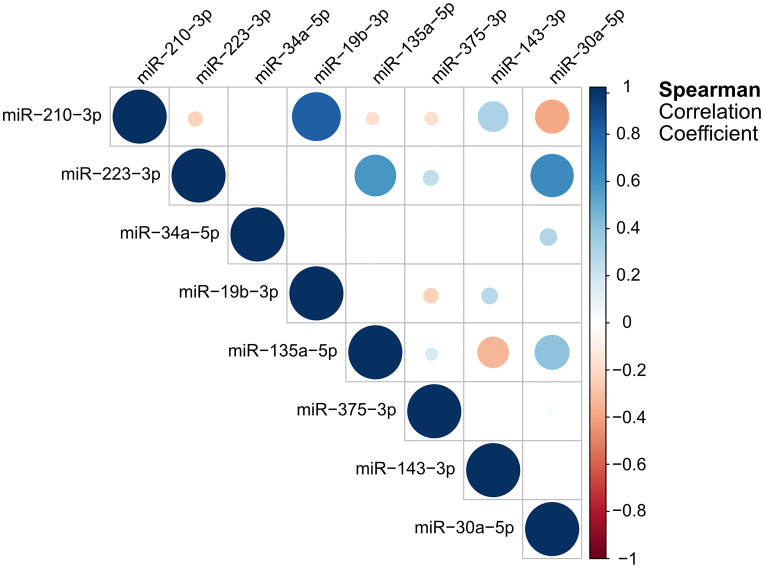
Spearman correlation analysis for the top miRNAs. Normalized dCq-values were used to perform pair-wise correlation between the top 8 miRNAs. Spearman correlation coefficients are visualized as heatmap, where the dot size and color represent the degree of positive or negative correlation.

To assess the redundancy between the top 8 validated miRNAs, we generated a Spearman correlation matrix and found that three miRNA pairs showed a high positive correlation, i.e., miR-19b-3p vs miR-210-3p, and miR-223-3p vs miR-30a-5p as well as vs miR-135a-5p. (Fig 3). All other pairs showed only moderate positive or negative association.

**Fig 4:**
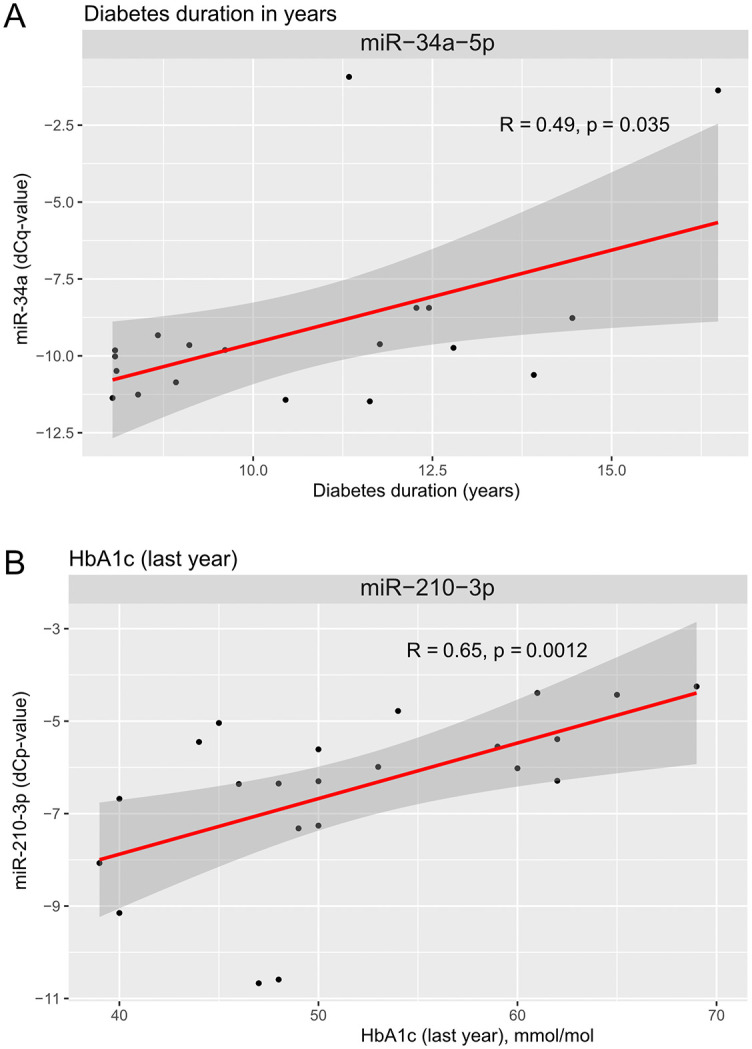
Association between circulating miRNA levels and clinical parameters. Two significant associations were identified. **(A)** Plasma levels of miR-34a-5p are associated with diabetes duration (n = 19 observations, 5 missing values below the limit of detection), and (**B**) the levels of miR-210-3p (n = 22 observations, 2 missing values below the limit of detection) are associated with the mean HbA1c level observed in the last year before sample collection for miRNA analysis.

The top 8 miRNAs (from Table 4) were further investigated in relation to age, BMI, HbA1c, diabetes duration and body composition parameters. Two miRNAs showed significant associations (Fig 4): miR-34a-5p levels were positively correlated with diabetes duration and miR-210-3p levels were positively correlated with HbA1c (average from the last year).

### Sex differences in circulating miRNA levels

To assess the associations between circulating miRNA levels and sex in young adolescents we performed a differential expression analysis between all male (n = 28) and female (n = 20) subjects from the T1D and control groups. This analysis identified 6 miRNAs with significant sex differences (adjusted p-value <0.1) (S1 Table) including miR-21-5p, miR-17-5p, miR-128-3p, miR-192-5p, miR-101-3p, miR-126-3p, which did not overlap with the top 8 miRNA candidates differentiating T1D from controls.

## Discussion

This case-control validation study of previously explored miRNAs [10], confirmed that miR-135a-5p and miR-223-3p are up-regulated in long-duration T1D in adolescents in comparison with healthy controls. In addition, six other miRNAs were differently regulated (adjusted p-value <0.05) in comparison with control individuals, of which miR-210-3p and miR-34a-5p showed significant associations with HbA1c levels (average from last year) and diabetes duration, respectively. Four additional miRNAs displayed adjusted p-values between 0.05 and 0.1 and therefore have slightly higher probability for false-discoveries. Therefore, the discussion of results will focus on miRNAs with the lowest adjusted p-values.

Several studies have identified miR-135a as a treatment target in diabetic nephropathy for renal fibrosis [25,26]. The observed upregulation of miR-135a in this validation study, as well as in our previous case-control discovery study [10], suggests a regulatory mechanism for miR-135a that could influence the development of renal fibrosis in T1D already during adolescence. MiR-135a is also of importance for reprogramming acinar cells into insulin producing cells [27], which implies a therapeutic potential for miR-135a in diabetes.

The higher cancer incidence among individuals with diabetes has been recognized for many decades [28,29]. A 2021 epidemiological analysis, comprising 313,907 matched individuals with and without diabetes, concluded a decline in vascular complications and that cancer now is the leading cause of diabetes-related death [30]. Well-powered studies have confirmed the link between cancer and cancer-related mortality in T1D, supporting further investigation of miRNAs as biomarkers and therapeutic targets [31,32]. Indeed, dysregulation of miR-135a can result in various forms of cancer, and it has been reported that miR-135a mediates cell proliferation and cancer progression through the MAPK and JAK2/STAT3 signaling pathways [33]. Several studies have suggested targeting miR-135a in cancer-related therapy to improve the outcome for individuals with cancer [34].

As recently reported, the plasma level of miR-223 was significantly higher in T1D compared to controls in our discovery study [10], which could be confirmed with this larger cohort of adolescents with T1D. The transcription of miR-223 is very high in hematopoietic cells, and is associated with autoimmunity, T1D, T2D, inflammation, obesity and diabetic microvascular complications [24,35]. The review by Gangwar et al. [36] demonstrated that miR-223 is associated with hypertension and atherosclerotic disease. A review on miR-223 and cancer development concluded that miR-223 has multiple established roles in hematopoiesis, inflammation, and several cancer types, where miR-223 functions as either an oncogenic or oncosuppressive miR [37].

MiR-34a-5p, a known biomarker of T2D and metabolic disease including metabolic dysfunction-associated steatotic liver disease (a.k.a. MASLD), was found to be lower in T1D but associated with diabetes duration. MiR-34a-5p, a member of the miR-34 family, is implicated in various cardiovascular pathologies, including myocardial infarction, heart failure, and atherosclerosis. It modulates key processes such as apoptosis, autophagy, inflammation, senescence, and remodeling by targeting signaling pathways like Smad4/TGF-β1, FOXO3/PUMA, Notch1/ETBR, PTEN/PI3K/SIRT1, and FOXM1/NRF2/HO-1 [38]. In line with this, Mone et al. [39] concluded that “miR-34 may be linked to diabetes and endothelial dysfunction”, proposing it as a potential biomarker of frailty in diabetic older adults, and highlighting its role in oxidative stress as a promising drug target for novel therapies.

The meta-analysis by Margaritis et al. [11] reported that miR-210-3p is higher in individuals with T1D, but associated to the average HbA1c level during the previous year. Their conclusion was, however, only based on two articles [40,41]. Osipova et al. [41], explored miR-210 in 68 individuals, aged 6–18 years, with a duration of T1D of at least 1 year (mean 5.0 years). They reported up-regulated miR-210 plasma levels in comparison with age- and sex-matched controls, which is in contrast to our findings. Nielsen et al. [40], investigated miR-210 in serum samples from newly diagnosed children with T1D and found up-regulated miR-210 levels. These opposing results, regarding up- or down-regulation in comparison with the current study, could be due to differences in T1D duration (between 8.0 to 16.5 years in this cohort), or differences in age. In addition, differences in the miRNA baseline levels between serum and EDTA-plasma samples, which are caused by miRNA release during blood clotting [42], could also contribute to such discrepancies.

The observed sex differences in circulating miRNA levels may reflect underlying biological distinctions between males and females during adolescence, including sex-hormone effects on gene regulation and immune function. Pubertal stage and circulating sex steroids can alter miRNA expression in multiple tissues and metabolic pathways, which may be reflected by serum miRNA profiles.

This study was powered based on previous findings and specifically designed to validate or refute the association of selected circulating miRNAs with T1D (Table 2). The long diabetes duration, between 8.0 to 16.5 years, in a narrow age span with registered metabolic data is a strength in this study. The study groups were well-matched regarding age and sex, and body composition did not differ between the groups. Strict exclusion criteria were applied, which resulted in a homogenous group of individuals with T1D. Validated state-of-the art RT-qPCR protocols [12,13] were used for analysis of circulating miRNAs. Known sources of methodological variability such as hemolysis, RT-qPCR inhibition, and lack of homogenous RNA recovery [43] were successfully controlled, which resulted in high-quality data with low analytical variability suitable for assessing miRNA variability in the context of T1D.

The present study also has limitations. A larger study group, including a wider age span and individuals with obesity, coeliac disease and hypothyroidism, i.e., conditions that frequently co-occur with T1D, would have been desirable to increase the generalizability to the target patient population. Considering that the research topic of TID and miRNAs is novel, with specific knowledge gaps and challenges, there might be yet unidentified miRNAs not included in this study.

## Conclusions

In this clinical case-control validation study, involving a well-matched cohort of young individuals with long-standing T1D (between 8.0 to 16.5 years), we demonstrate that the plasma levels of miR-223-3p and miR-135a-5p are elevated. Given the established links of miR-223-3p to cardiovascular and inflammatory disease, and miR-135a-5p to cancer, these findings suggest that both miRNAs may serve as promising biomarkers for the early identification of patients at increased risk of diabetes-related complications and malignancies. Future larger-scale, prospective studies incorporating a broader validation of the proposed miRNA biomarkers in independent study populations is still needed to generate sufficient scientific evidence for their use in clinical practice. Particular attention should be paid to minimizing pre-analytical and analytical variability in sample collection and processing to ensure the reliability of miRNA measurements in broader clinical applications.

## Supporting information

S1 Fig RT-qPCR and sample quality control. (A) The recovery of exogenous spike-in controls added prior to the RNA extraction or (B) during reverse transcription and qPCR amplification was assessed and visualized as dot plots. (C) Hemolysis was assessed on the basis of the ratio between miR-451a and miR-23a.
(TIF)

S1 Table Gender differences in circulating miRNA levels. (DOCX)
