# Supplementary figures and images for: Independent validation of circulating microRNAs as biomarkers in a case-control study of adolescents with type 1 diabetes for more than 8 years
Source: PLoS One. 2026 Feb 23;21(2):e0343117. doi: 10.1371/journal.pone.0343117 (PMC12928441; doi:10.1371/journal.pone.0343117)

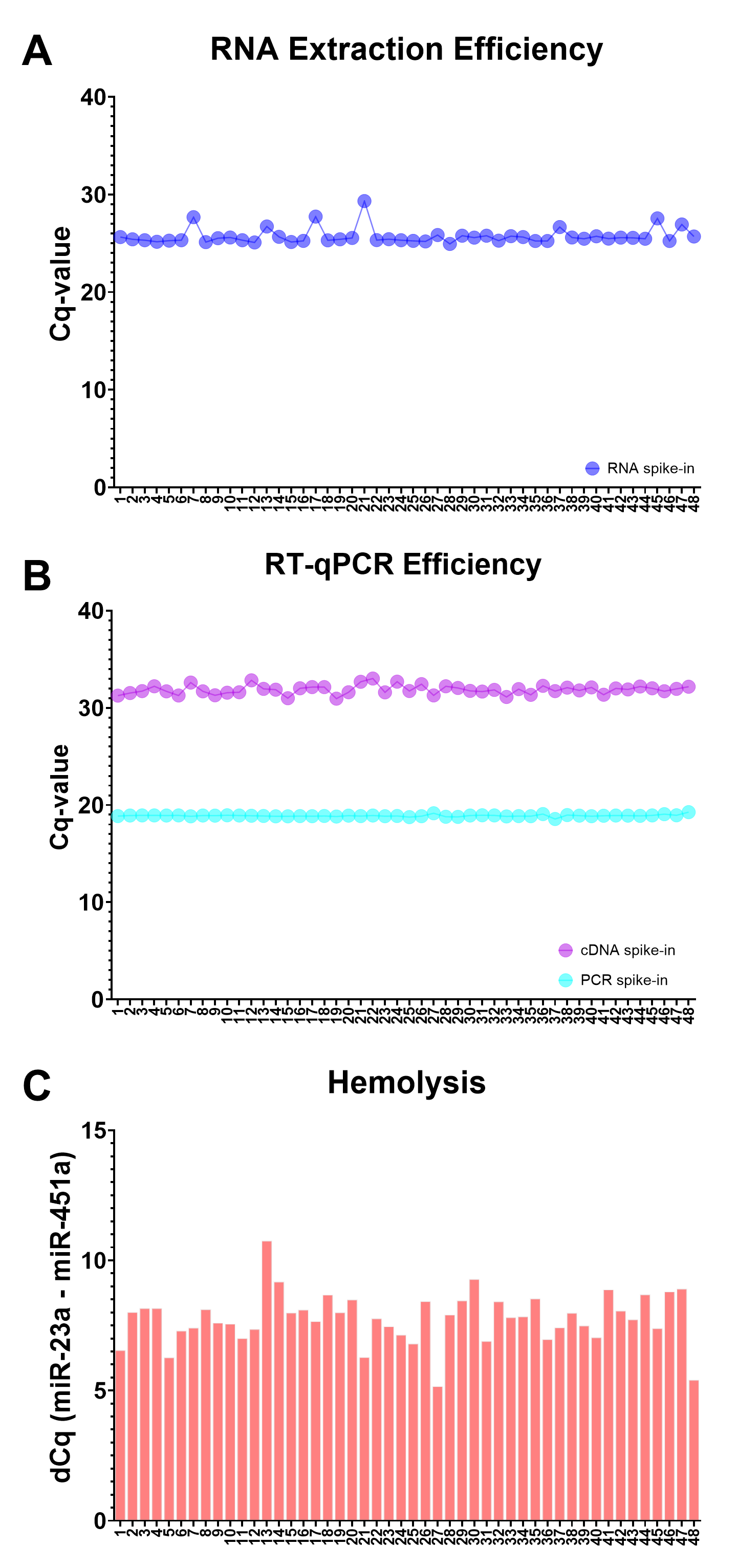

Supplement: S1 Fig — (A) The recovery of exogenous spike-in controls added prior to the RNA extraction or (B) during reverse transcription and qPCR amplification was assessed and visualized as dot plots. (C) Hemolysis was assessed on the basis of the ratio between miR-451a and miR-23a. (TIF) [file pone.0343117.s001.tif]
