# Supplementary material for: Independent validation of circulating microRNAs as biomarkers in a case-control study of adolescents with type 1 diabetes for more than 8 years
Source: PLoS One. 2026 Feb 23;21(2):e0343117. doi: 10.1371/journal.pone.0343117 (PMC12928441; doi:10.1371/journal.pone.0343117)
Supplement: S1 Table — (DOCX) [file pone.0343117.s002.docx]

**Title:** Independent validation of circulating microRNAs as biomarkers in a case-control study of adolescents with type 1 diabetes for more than 8 years

**Authors:** Diana Swolin-Eide et al.

**Supplementary Table 1.** Gender differences in circulating miRNA levels

| **miRNA-ID** | **AUC**  **(from ROC)** | ***P*-value**  **(Mann-Whitney)** | **Adjusted**  ***p*-value (BH)** |
| --- | --- | --- | --- |
| **miR-21-5p** | **0.77** | **0.002** | **0.021** |
| **miR-17-5p** | **0.74** | **0.005** | **0.059** |
| **miR-128-3p** | **0.72** | **0.010** | **0.073** |
| **miR-192-5p** | **0.73** | **0.007** | **0.078** |
| **miR-101-3p** | **0.72** | **0.010** | **0.086** |
| **miR-126-3p** | **0.71** | **0.012** | **0.097** |
| miR-29b-3p | 0.70 | 0.019 | 0.15 |
| miR-215-5p | 0.70 | 0.018 | 0.16 |
| miR-181b-5p | 0.65 | 0.07 | 0.30 |
| miR-143-3p | 0.62 | 0.18 | 0.40 |
| miR-210-3p | 0.61 | 0.21 | 0.41 |
| miR-19b-3p | 0.61 | 0.21 | 0.50 |
| miR-146a-5p | 0.64 | 0.09 | 0.66 |
| miR-34a-5p | 0.57 | 0.44 | 0.66 |
| miR-30a-5p | 0.55 | 0.57 | 0.74 |
| miR-135a-5p | 0.58 | 0.35 | 0.80 |
| miR-1246 | 0.59 | 0.32 | 0.85 |
| miR-122-5p | 0.55 | 0.54 | 0.90 |
| miR-495-3p | 0.57 | 0.42 | 0.93 |
| miR-223-3p | 0.51 | 0.89 | 0.96 |
| miR-410-3p | 0.56 | 0.50 | 0.97 |
| miR-124-3p | 0.51 | 0.90 | 0.99 |
| miR-375-3p | 0.50 | 0.99 | 1.00 |

Bold font indicate adjusted *p*-values of <0.1.

*AUC* area under the curve, *BH* Benjamini-Hochberg, *miRNA* microRNA, *ROC* receiver operator characteristic.
